# Developmental milestones and daily living skills in individuals with Angelman syndrome

**DOI:** 10.1186/s11689-024-09548-7

**Published:** 2024-06-15

**Authors:** Anjali Sadhwani, Sonya Powers, Anne Wheeler, Hillary Miller, Sarah Nelson Potter, Sarika U. Peters, Carlos A. Bacino, Steven A. Skinner, Logan K. Wink, Craig A. Erickson, Lynne M. Bird, Wen-Hann Tan

**Affiliations:** 1 Department of Psychiatry and Behavioral Services, Boston Children’s Hospital, Harvard Medical School, Boston, MA USA; 2 Department of Psychiatry and Behavioral Services, Boston Children’s Hospital, 300 Longwood Avenue, Boston, MA 02115 USA; 3 RTI International, Research Triangle Park, NC, USA; 4 Harvard T.H. Chan School of Public Health, Boston, MA USA; 5 Vanderbilt University School of Medicine, Nashville, TN USA; 6 Kleberg Genetics Clinic, Texas Children’s Hospital, Baylor College of Medicine, Houston, TX USA; 7 Greenwood Genetic Center, Greenwood, SC USA; 8 Division of Child Psychiatry, Cincinnati Children’s Hospital Medical Center, Cincinnati, USA; 9 University of California San Diego and Rady Children’s Hospital, San Diego, CA USA; 10 Edmentum, Minneapolis, MN USA; 11 Aetna, Hartford, CT USA; 12 Talkiatry Management Services, LLC, New York, USA; 13 Division of Genetics and Genomics, Boston Children’s Hospital, Harvard Medical School, Boston, United States

**Keywords:** Child development, Developmental disabilities, Intellectual disability, Activities of Daily Living

## Abstract

**Background:**

Angelman syndrome (AS) is a neurodevelopmental disorder associated with severe global developmental delay. However, the ages at which different developmental skills are achieved in these individuals remain unclear. We seek to determine the probability and the age of acquisition of specific developmental milestones and daily living skills in individuals with AS across the different molecular subtypes, viz. class I deletion, class II deletion, uniparental disomy, imprinting defect, and *UBE3A* variants.

**Methods:**

Caregivers participating in a longitudinal multicenter Angelman Syndrome Natural History Study completed a questionnaire regarding the age at which their children achieved specific developmental milestones and daily living skills. The Cox Proportional Hazard model was applied to analyze differences in the probability of achievement of skills at various ages among five molecular subtypes of AS.

**Results:**

Almost all individuals, regardless of molecular subtype, were able to walk with support by five years of age. By age 15, those with a deletion had at least a 50% probability of acquiring 17 out of 30 skills compared to 25 out of 30 skills among those without a deletion. Overall, fine and gross motor skills such as holding and reaching for small objects, sitting, and walking with support were achieved within a fairly narrow range of ages, while toileting, feeding, and hygiene skills tend to have greater variability in the ages at which these skills were achieved. Those without a deletion had a higher probability (25–92%) of achieving daily living skills such as independently toileting and dressing compared to those with a deletion (0–13%). Across all molecular subtypes, there was a low probability of achieving independence in bathing and brushing teeth.

**Conclusion:**

Individuals with AS without a deletion are more likely to achieve developmental milestones and daily living skills at an earlier age than those with a deletion. Many individuals with AS are unable to achieve daily living skills necessary for independent self-care.

**Supplementary Information:**

The online version contains supplementary material available at 10.1186/s11689-024-09548-7.

## Background

Angelman syndrome (AS) is a neurodevelopmental disorder characterized by severe intellectual disability, minimal or absent speech, ataxia, epilepsy, and sleep disturbances [1–3]. The prevalence of AS is approximately 1 in 22,000 to 1 in 52,000 [4–8]. AS is caused by lack of expression in neurons of the maternally-inherited copy of the ubiquitin-protein ligase E3A gene (*UBE3A*) on chromosome 15q11.2 [9–11] due to one of four mechanisms: deletion on the maternal copy of chromosome 15q11.2q13.1 (approximately 65–70% of individuals with AS), paternal uniparental disomy (UPD) (approximately 10%), an imprinting defect (ImpD) that results in the maternal allele being silenced in neurons (approximately 5–10%), and a pathogenic variant in the maternally-inherited *UBE3A* allele (about approximately 10–15%). The deletion subtypes can be further classified into: (a) class I with a 5.9 Mb deletion (40%), (b) class II with a 5.0 Mb deletion (50–55%), and (c) atypical deletions, which are smaller than class II or larger than class I (5–10%) [1, 12–14]. Those with a deletion are classified as “deletion-positive” while those with all other molecular subtypes are classified as “deletion-negative”.

Previous studies have found that individuals with AS are significantly delayed across all domains of development, but they do make slow developmental gains over time [15–23]. The severity of the developmental delay varies by molecular subtype; those with a deletion have more severe developmental delay than those without a deletion [16, 17, 19–25]..

The likelihood and the rate of acquiring the various developmental milestones are dependent on the molecular subtype of the child. Reports on the ages at which specific milestones are achieved are limited and have focused mainly on gross motor skills [16, 22, 26]. For example, Lossie et al. found that individuals with a deletion achieve motor milestones at a later age compared to those without deletion (sitting: 1.3 years versus 0.7–1.0 years; walking independently: 4.6 years versus 2.5–2.9 years) [26].

Understanding developmental milestones in AS is important both for clinical management and to determine the efficacy of future potential treatments. These milestones can help clinicians provide caregivers with more accurate evidence-based prognoses of developmental outcomes based on molecular subtype. If a child with AS is progressing more slowly than expected, clinicians should consider potential medical complications that might be affecting the child’s development (such as subclinical seizures or non-convulsive status epilepticus) or a second diagnosis that might explain the deviation from the anticipated developmental trajectory. More realistic developmental goals can also be set by the child’s educational intervention team using these milestones. Attainment of milestones earlier than expected in clinical trials of compounds that target neurodevelopment would suggest that the investigational product might be effective.

Using data from a large-scale longitudinal multicenter natural history study, we sought to determine the ages at which various developmental milestones and daily living skills were acquired in individuals with AS and how age of acquisition varies across different molecular etiologies.

## Methods

### Study population

Participants in this study were from the AS Natural History study (ClinicalTrials.gov identifier: NCT00296764). Inclusion criteria included: a confirmed molecular diagnosis of AS, absence of other co-morbid developmental disorders (e.g., severe prematurity or an additional genetic diagnosis), and age between one day and 60 years. We excluded individuals with a deletion of an atypical or unknown size, and individuals without developmental milestone data. Participants were evaluated annually at one of six study sites: Rady Children’s Hospital San Diego, Texas Children’s Hospital, Greenwood Genetic Center, Boston Children’s Hospital, Vanderbilt University Medical Center, and Cincinnati Children’s Hospital Medical Center. The study was approved by the institutional review boards at each study site.

### Measure

At each study visit, parents completed a clinician-developed questionnaire [supplementary material] on whether their child had acquired specific developmental milestones in the domains of gross and fine motor development, and receptive and expressive language, and if so, the ages at which those skills were acquired. However, in this study, only a subset of developmental milestones was analyzed. The specific gross motor, fine motor, and expressive language skills that were analyzed were those that met the following criteria: (a) were meaningful to the parents, (b) impacted quality of life and daily functioning, (c) representative of development in each domain and (d) the study team believed that parents could recall accurately. Gross motor milestones included skills such as sitting unsupported, commando crawl, four-point crawl, pulling to stand, walking with support, and walking independently. Fine motor milestones included skills such as reaching for an object, holding an object, transferring an object from one hand to another, and using a pincer grasp. Expressive language included skills such as gesturing or pointing for wants, use of manual signs (including American Sign Language and enhanced natural gestures), and use of single words. Questions on daily living skills (toileting, dressing, washing hands, bathing, brushing teeth, and feeding) were introduced later in the study, so only a subset of the sample answered these questions.

### Data cleaning

Analysis of a given skill by a participant was excluded if: (i) acquisition of the skill was marked “unknown”; (ii) participants were classified as having acquired the skill, but the age of acquisition was missing or unknown; (iii) the age of skill acquisition was greater than the chronological age at the visit; and (iv) age of skill acquisition was inconsistently reported at different study visits. Application of these data cleaning rules resulted in little data loss (less than 10% for most skills).

### Statistical analysis

The Cox Proportional Hazard (CPH) Model was used to compare the probability that individuals with AS would achieve selected developmental milestones and daily living skills across five molecular subtypes, i.e., class I deletion, class II deletion, UPD, ImpD, and *UBE3A* pathogenic variants. The CPH is a regression model well suited to analyzing data where time to an event is the outcome of interest. When an individual fails to achieve the event during the observation window, the data are considered “censored”, indicating that it is possible that the participant could achieve the skill in the future beyond the temporal observation window of this study.

Cumulative hazard curves were created to visually compare the probability of achieving the 30 selected developmental milestones and daily living skills (six gross motor, four fine motor, three expressive language, three toileting, five dressing, six hygiene, and three feeding skills). The hazard rate indicates the probability of achieving a given skill at a particular age. The hazard rate for participants with a class I deletion, UPD, ImpD, or a *UBE3A* pathogenic variant was compared to the reference hazard rate of the participants with the most common molecular subtype in our sample (i.e., class II deletion), which had the largest sample size, by testing the ratio of the hazard rates for significance. The ratio of two hazard rates is called the hazard ratio. Hazard ratios greater than 1 indicate a higher probability of achieving the skill compared to participants with a class II deletion, whereas hazard ratios less than 1 indicate a lower probability of achieving the skill compared to participants with the reference subtype. Hazard ratios with *p*-values less than 0.05 were considered significant.

The ages at which there was a 5% and a 95% probability of acquiring a given skill were identified as a way to capture the range in the ages at which each skill was likely to be achieved. Graphical results were plotted for participants within each molecular subtype, truncated at 15 years of age because little to no change in development was observed beyond this point. Moreover, approximately 90% of the observations included in these analyses were from individuals younger than 15 years of age.

An assumption of the CPH model is that the hazard ratio is constant across time. If the assumption holds, the relative risk of an event is constant over time. The cox.zph R function was used to test for statistically significant violations of the proportional hazard assumption. In addition, recall duration was computed as the time interval between when the skill was achieved and when it was first reported and included as a model covariate, with significant results indicating possible recall bias. Sex was also included as a covariate to determine whether there were any differences between males and females in the probability of skill acquisition. All significance tests were evaluated using an alpha level of *p* < 0.05.

## Results

### Sample characteristics

Caregivers of 261 participants (Table 1) completed the developmental milestones questionnaire. Twenty-eight percent of the participants had a class I deletion, 40% had a class II deletion, 13% had *UBE3A* pathogenic variants, 8% had ImpD, and 11% had UPD. All the *UBE3A* variants were loss-of-function variants [27]. The number of visits per participant ranged from one to nine (*Mean*: 3.7, *SD*: 2.3). Age at baseline visit ranged from 0.4 years to 40.6 years (*Mean*: 5.6, *SD*: 5.3). There were no significant differences in the number of visits, age at the first (baseline) visit, or age at final visit among participants with different molecular subtypes. A subset of 211 participants completed the daily living skills questionnaire and on average, they were slightly older at baseline (*Mean*: 6.1, *SD*: 5.7) and had more visits (*Mean*: 4.1, *SD*: 2.3) compared to those who did not complete this questionnaire.

**Table 1:** Sample Characteristics

	**Class I Deletion**	**Class II Deletion**	***UBE3A* Variant**	**Imprinting Defect**	**Uniparental Disomy**	**Overall**	***p***
	**(*N* = 72)**	**(*N* = 105)**	**(*N* = 33)**	**(*N* = 22)**	**(*N* = 29)**	***N* = 261**	
	**28%**	**40%**	**13%**	**8%**	**11%**		
**Gender**							
Male	32 (44%)	54 (51%)	19 (58%)	12 (55%)	13 (45%)	130 (50%)	NS
Female	40 (56%)	51 (49%)	14 (42%)	10 (45%)	16 (55%)	131 (51%)	
**Age at Baseline (years)**							
Mean (*SD*)	5.7 (7.1)	5.2 (4.8)	5.7 (3.9)	5.9 (4.2)	6.7 (4.3)	5.6 (5.3)	NS
Range	(0.9–40.6)	(1.1–26.8)	(0.4–14.6)	(2.3–21.0)	(2.1–20.8)	(0.4–40.6)	
**Age at Final Visit (years)**							
Mean (*SD*)	8.6 (7.6)	8.0 (5.5)	8.2 (4.1)	9.7 (4.8)	8.8 (4.4)	8.4 (5.9)	NS
Range	(1.1–40.6)	(1.1–30.4)	(1.4–18.6)	(3.6–26.7)	(2.5–24.0)	(1.1–40.6)	
**Number of Visits**							
Mean (*SD*)	3.8 (2.4)	3.8 (2.2)	3.5 (2.5)	4.7 (2.4)	3 (1.4)	3.7 (2.3)	NS
Range	(1–9)	(1–8)	(1–8)	(1–8)	(1–5)	(1–9)	
**History of Seizures**							
Yes	51 (71%)	69 (66%)	9 (27%)	7 (32%)	16 (55%)	152 (58%)	< .0001
No	7 (10%)	8 (8%)	12 (36%)	11 (50%)	7 (24%)	45 (17%)	
Unknown	14 (19%)	28 (26%)	12 (36%)	4 (18%)	6 (21%)	64 (25%)	

Note. Age at Baseline is the age of the patient when the first set of data was collected; Age at Final Visit is the age of the patient when the last set of data was collected

### Probability of achieving different developmental milestones

**Fig. 1:**
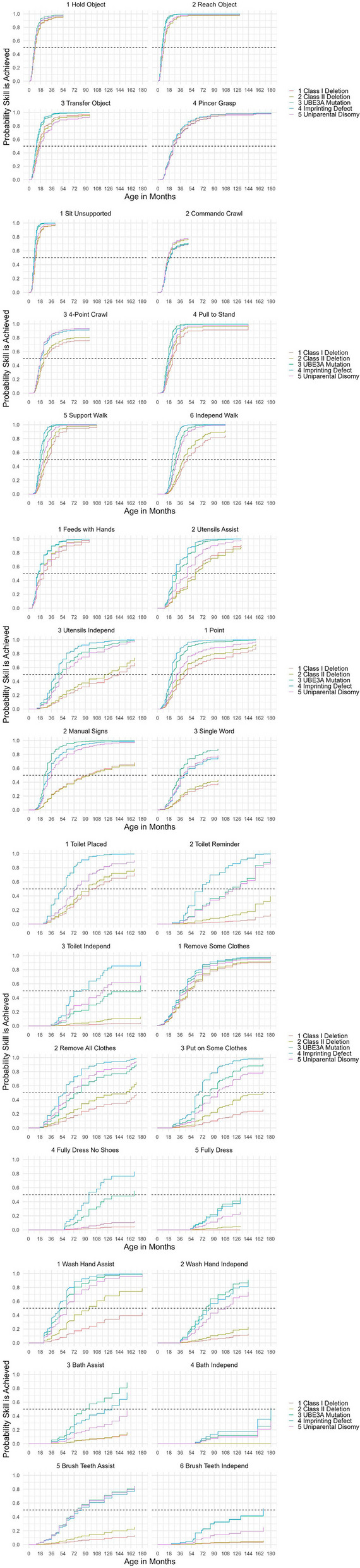
Probability of Skill Achievement for Critical Developmental Milestones and Daily Living Skills by Molecular Subtype Note. Dashed lines indicate point on the curve associated with a 50% probability of achieving the skill. Higher probabilities indicate that more individuals with AS are expected to achieve the skill. Steeper curves indicate that individuals tend to achieve the skill in a relatively narrow age range whereas flatter curves indicate a wide range of ages where individuals achieve the skill

The probability of an individual achieving a specific skill at a given age for various developmental milestones and daily living skills is depicted in Fig. 1. We found that some developmental milestones in the fine and gross motor domains were more likely to be achieved compared to other types of skills. For example, almost all individuals, regardless of molecular subtype, were able to walk with support by age five years. In contrast, for some molecular subtypes certain daily living skills were more difficult to achieve (i.e., probabilities remained low across the age range and were achieved by fewer than 95% of the subgroup at 15 years of age). For example, the probability that an individual can brush his/her teeth with assistance by 15 years of age was 13% for participants with class I deletion compared to 85% for those with a *UBE3A* pathogenic variant. Other daily living skills such as brushing teeth independently and bathing independently had a low probability of achievement by age 15 across molecular subtypes (Fig. 1).

**Fig. 2:**
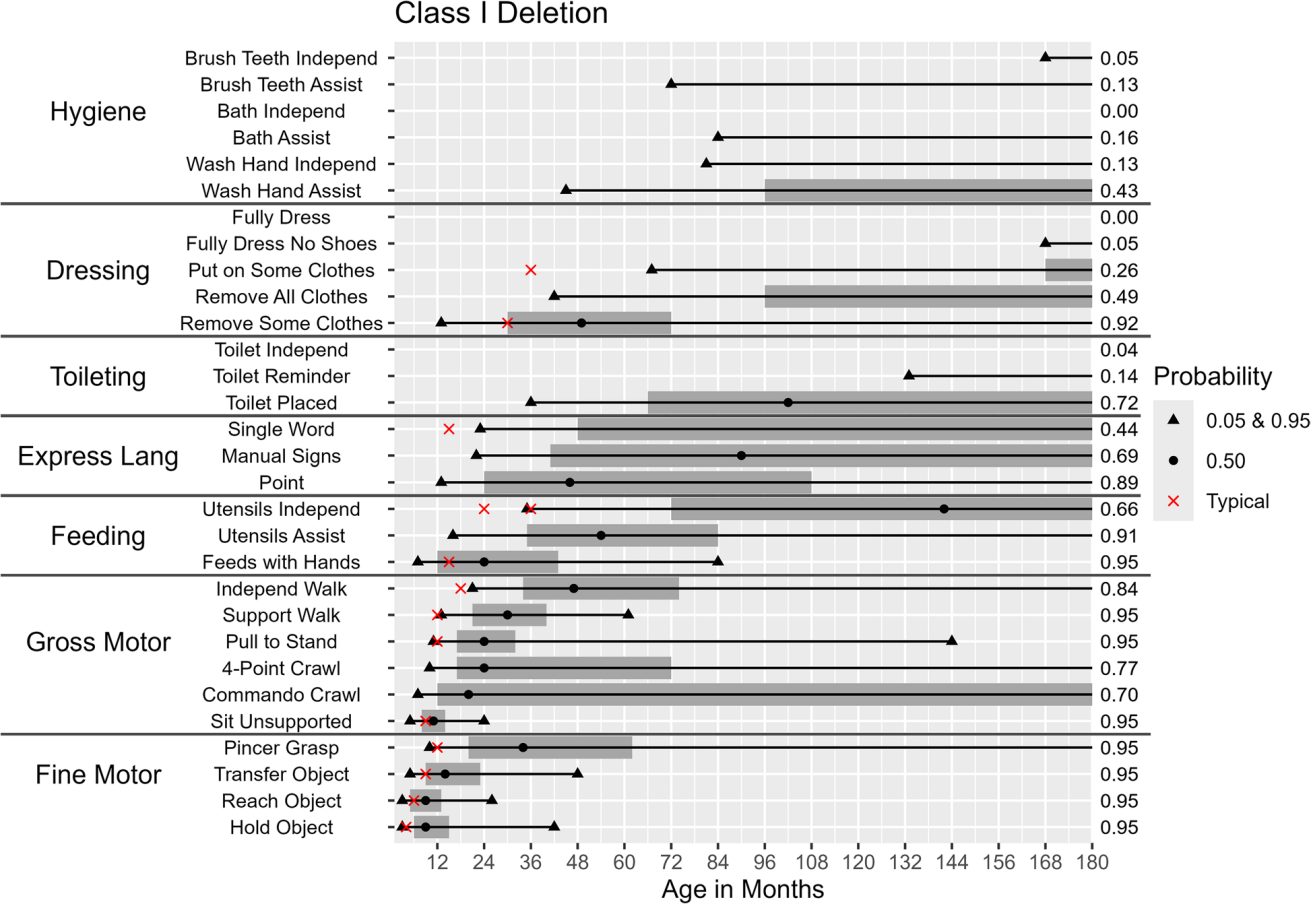
Probability of Skill Achievement in Class I Deletion From left to right, the first black triangle represents a probability of 0.05, the beginning of the shaded gray bar represents a probability of 0.25, the black circle represents a probability of 0.50, the end of shaded gray bar on the right represents a probability of 0.75, and a second black triangle on the right represents a probability of 0.95. The red X indicates the age at which the milestone is achieved by ≥ 75% children in the general population. For the feeding skill of independent utensil use ("Utensils Independ") the first red X indicates use of a spoon, the second red X indicates use of a fork. The axis on the right indicates the probability of skill achievement. This value is either 0.95, or the probability of skill achievement at 15 years of age in cases where the probability did not reach 0.95

**Fig. 3:**
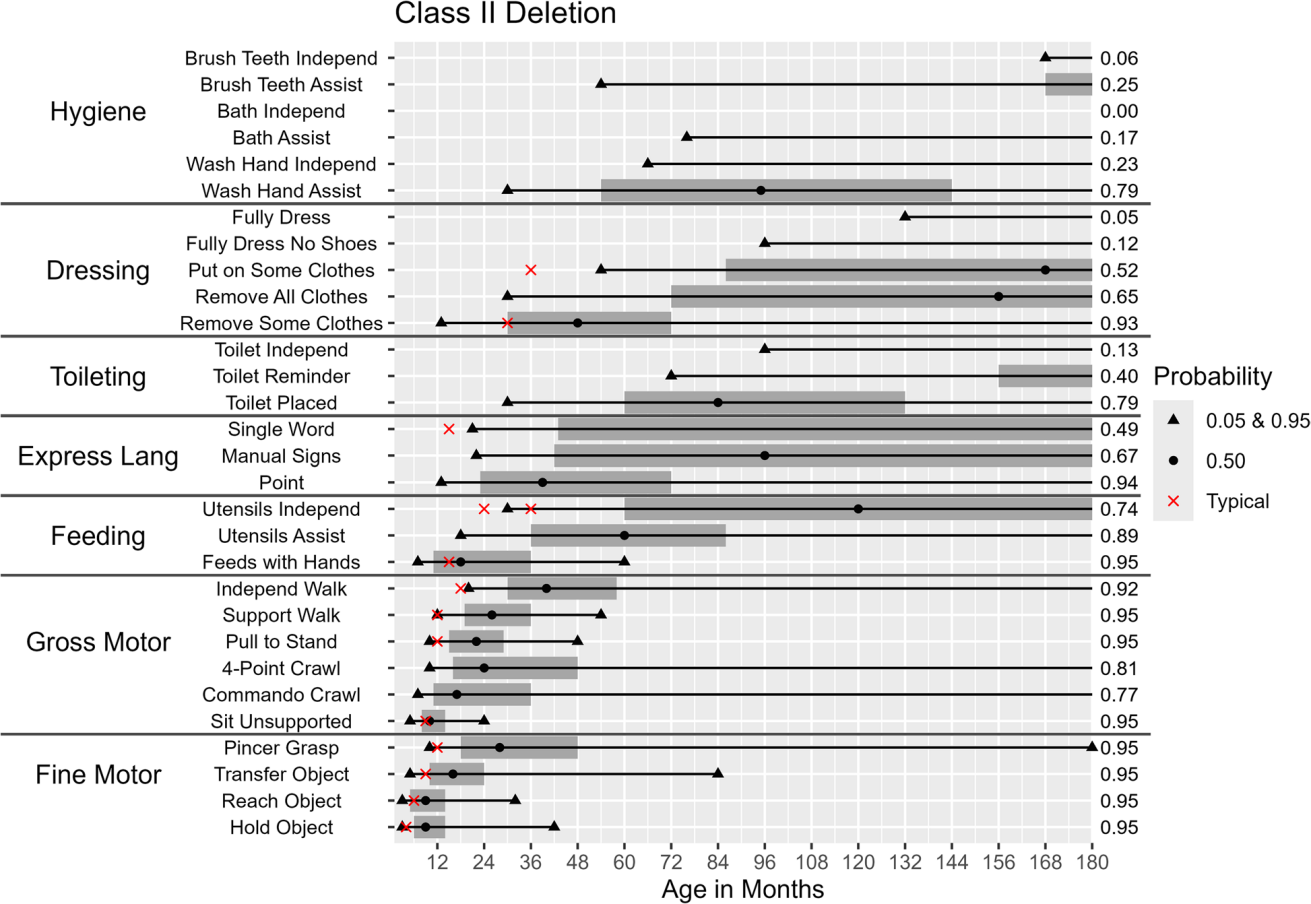
Probability of Skill Achievement in Class II Deletion From left to right, the first black triangle represents a probability of 0.05, the beginning of the shaded gray bar represents a probability of 0.25, the black circle represents a probability of 0.50, the end of shaded gray bar on the right represents a probability of 0.75, and a second black triangle on the right represents a probability of 0.95. The red X indicates the age at which the milestone is achieved by ≥ 75% children in the general populationFor the feeding skill of independent utensil use ("Utensils Independ") the first red X indicates use of a spoon, the second red X indicates use of a fork. The axis on the right indicates the probability of skill achievement. This value is either 0.95, or the probability of skill achievement at 15 years of age in cases where the probability did not reach 0.95

**Fig. 4:**
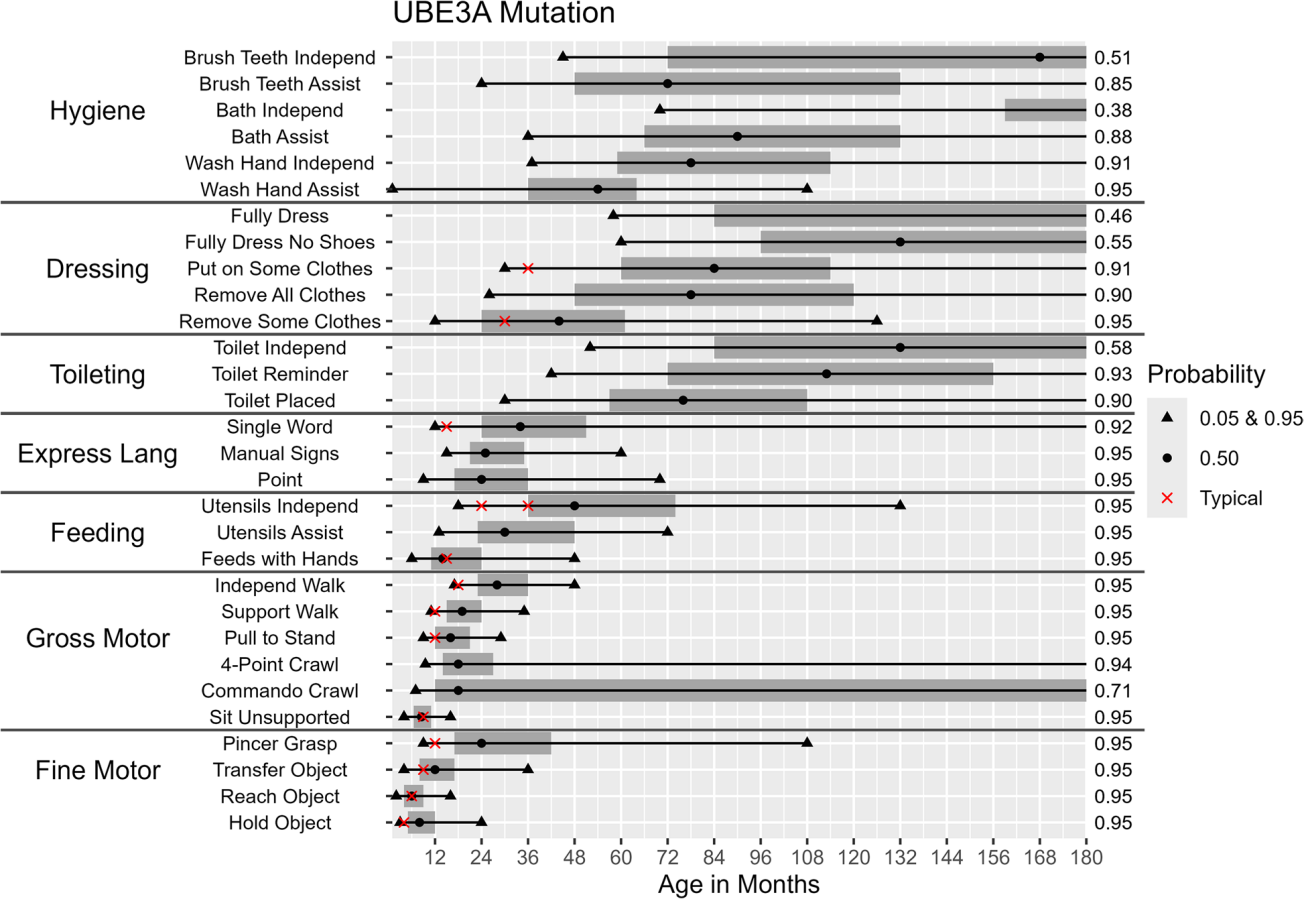
Probability of Skill Achievement in *UBE3A* Mutation From left to right, the first black triangle represents a probability of 0.05, the beginning of the shaded gray bar represents a probability of 0.25, the black circle represents a probability of 0.50, the end of shaded gray bar on the right represents a probability of 0.75, and a second black triangle on the right represents a probability of 0.95. The red X indicates the age at which the milestone is achieved by ≥ 75% children in the general populationFor the feeding skill of independent utensil use ("Utensils Independ") the first red X indicates use of a spoon, the second red X indicates use of a fork. The axis on the right indicates the probability of skill achievement. This value is either 0.95, or the probability of skill achievement at 15 years of age in cases where the probability did not reach 0.95

**Fig. 5:**
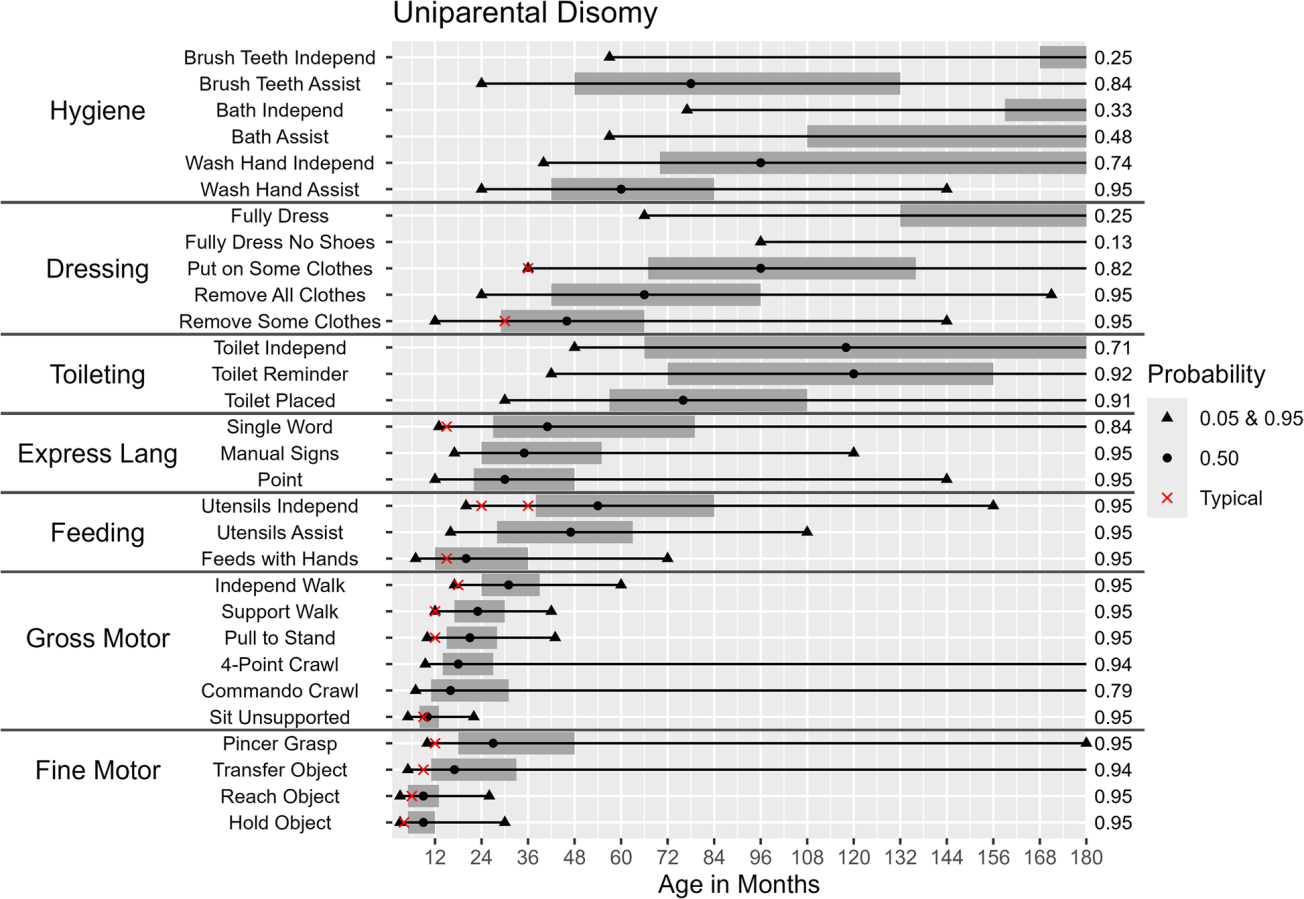
Probability of Skill Achievement in Uniparental Disomy From left to right, the first black triangle represents a probability of 0.05, the beginning of the shaded gray bar represents a probability of 0.25, the black circle represents a probability of 0.50, the end of shaded gray bar on the right represents a probability of 0.75, and a second black triangle on the right represents a probability of 0.95. The red X indicates the age at which the milestone is achieved by ≥ 75% children in the general populationFor the feeding skill of independent utensil use ("Utensils Independ") the first red X indicates use of a spoon, the second red X indicates use of a fork. The axis on the right indicates the probability of skill achievement. This value is either 0.95, or the probability of skill achievement at 15 years of age in cases where the probability did not reach 0.95

**Fig. 6:**
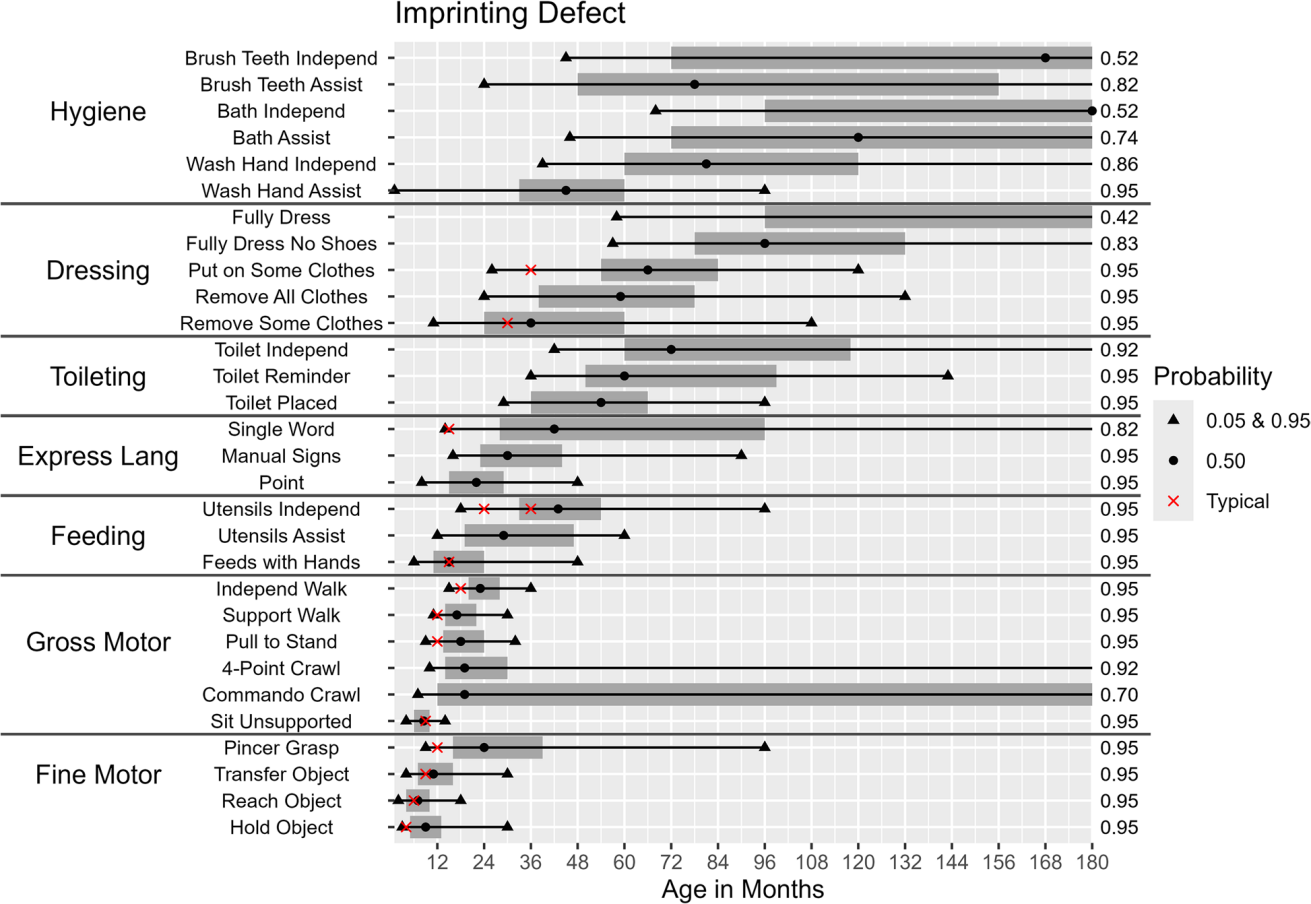
Probability of Skill Achievement in Imprinting Defect From left to right, the first black triangle represents a probability of 0.05, the beginning of the shaded gray bar represents a probability of 0.25, the black circle represents a probability of 0.50, the end of shaded gray bar on the right represents a probability of 0.75, and a second black triangle on the right represents a probability of 0.95. The red X indicates the age at which the milestone is achieved by ≥ 75% children in the general populationFor the feeding skill of independent utensil use ("Utensils Independ") the first red X indicates use of a spoon, the second red X indicates use of a fork. The axis on the right indicates the probability of skill achievement. This value is either 0.95, or the probability of skill achievement at 15 years of age in cases where the probability did not reach 0.95

Figures 2, 3, 4, 5 and 6 illustrate the ages at which there is a 5%, 50%, and 95% probability of achieving the selected developmental milestones and daily living skills among participants of each molecular subtype. For each skill with relevant comparative data, we have also indicated the age at which at least 75% of children in the general population achieve the milestone [28]. The probability of achieving these skills was found to be highly dependent on molecular subtype. By age 15, those with a deletion had at least a 50% probability of acquiring 17 out of 30 skills (Figs. 2 & 3) compared to 25 out of 30 skills among those with deletion-negative subtypes (Figs. 4, 5 and 6). Compared to deletion-negative participants, deletion-positive individuals had a lower probability of achieving self-care skills such as brushing teeth independently and hand washing independently by age 15. For example, the probability of independent hand washing was 91% by age 15 for those with *UBE3A* pathogenic variants (Fig. 4), 74% for those with UPD (Fig. 5), but only 13% for those with a class I deletion (Fig. 2).

Differences between molecular subtypes in the probability of skill acquisition were tested for significance using hazard ratios. Table 2 provides hazard ratios for the selected developmental milestones and daily living skills. The ratios comparing the class I and class II deletion groups were only significant for two out of the 30 skills (“putting on some clothes” [hazard ratio = 0.40, *p* < 0.05] and “washing hands with assistance” [hazard ratio = 0.37, *p* < 0.01]).

**Table 2:** Hazard Ratios for Developmental Skills by Molecular Subtype

	SUBTYPES^1^			
	**Class I Deletion**	***UBE3A* Mutation**	**Imprinting Defect**	**Uniparental Disomy**
**Gross** **Motor Skills**^2^				
Unsupported Sit	0.97 (0.70–1.33)	1.88 (1.23–2.88)*	2.19 (1.33–3.58)*	1.21 (0.78–1.88)
Commando Crawl	0.81 (0.57–1.17)	0.85 (0.52–1.42)	0.83 (0.45–1.53)	1.06 (0.65–1.72)
Four-Point Crawl	0.88 (0.61–1.26)	1.65 (1.06–2.56)*	1.50 (0.90–2.51)	1.66 (1.06–2.60)*
Pulls to Stand	0.74 (0.53–1.03)	2.17 (1.42–3.32)*	1.60 (0.98–2.60)	1.12 (0.72–1.75)
Walks with Support	0.77 (0.56–1.07)	2.35 (1.52–3.63)*	3.40 (2.10–5.49)*	1.48 (0.96–2.29)
Walks Independently	0.75 (0.52–1.09)	2.89 (1.88–4.45)*	5.80 (3.51–9.59)*	2.19 (1.39–3.46)*
**Fine Motor Skills**				
Holds Small Object^2^	0.97 (0.70–1.35)	1.35 (0.85–2.12)	1.15 (0.68–1.96)	1.17 (0.73–1.87)
Reaches for Object	1.12 (0.80–1.57)	1.90 (1.21–2.98)*	1.67 (1.00–2.79)	1.11 (0.69–1.78)
Transfers Object Hand to Hand	1.14 (0.81–1.59)	1.71 (1.07–2.73)*	1.93 (1.11–3.36)*	0.85 (0.49–1.48)
Uses Pincer Grasp	0.79 (0.55–1.14)	1.17 (0.74–1.86)	1.29 (0.78–2.14)	1.02 (0.64–1.63)
**Expressive Language Skills**				
Gestures/Points for Wants	0.82 (0.56–1.20)	2.20 (1.40–3.46)*	3.14 (1.90–5.20)*	1.35 (0.85–2.14)
Use of Manual Signs	1.04 (0.64–1.68)	6.81 (4.07–11.38)*	4.49 (2.60–7.75)*	3.51 (2.11–5.82)*
Single Words	0.87 (0.49–1.54)	3.77 (2.14–6.63)*	2.55 (1.38–4.70)*	2.75 (1.56–4.85)*
**Toileting**^3^				
Uses Toilet When Placed	0.75 (0.43–1.32)	1.44 (0.76–2.72)	3.72 (2.03–6.83)*	1.44 (0.77–2.68)
Uses Toilet When Reminded	0.28 (0.06–1.30)	4.98 (2.01–12.36)*	13.30 (5.32–33.25)*	4.55 (1.78–11.61)*
Uses Toilet Without Reminder^4^	0.32 (0.04–2.77)	6.06 (1.96–18.71)*	17.45 (5.84–52.17)*	8.83 (2.94–26.55)*
**Dressing**				
Can Remove Some Clothes	0.88 (0.60–1.31)	1.29 (0.81–2.07)	1.44 (0.86–2.40)	1.16 (0.69–1.96)
Can Remove All Clothes	0.61 (0.31–1.20)	2.13 (1.16–3.94)*	4.20 (2.22–7.94)*	2.73 (1.46–5.13)*
Can Put on Some Clothes	0.40 (0.16–1.00)*	3.16 (1.66–6.00)*	5.94 (3.01–11.74)*	2.24 (1.05–4.78)*
Can Fully Dress Except Shoes	0.38 (0.04–3.38)	5.86 (1.71–20.03)*	12.86 (3.96–41.83)*	0.98 (0.11–8.79)
Can Fully Dress Including Shoes	†	11.29 (2.34–54.39)*	10.03 (1.94–51.95)*	5.35 (0.89–32.07)
**Hygiene**				
Washes Hands With Assistance	0.37 (0.18–0.76)*	3.15 (1.82–5.46)*	4.18 (2.25–7.75)*	2.34 (1.31–4.19)*
Washes Hands Independently	0.51 (0.14–1.90)	9.00 (3.97–20.38)*	7.46 (3.17–17.54)*	4.99 (2.05–12.12)*
Bathes With Assistance	0.94 (0.22–3.95)	11.85 (4.21–33.37)*	7.38 (2.38–22.88)*	3.62 (1.05–12.54)*
Bathes Independently	†	†	†	†
Brushes Teeth With Assistance	0.48 (0.13–1.74)	6.26 (2.83–13.86)*	5.65 (2.47–12.92)*	6.08 (2.68–13.83)*
Brushes Teeth Independently	0.77 (0.07–8.52)	11.94 (2.53–56.36)*	12.13 (2.44–60.25)*	4.70 (0.78–28.20)
**Feeding**				
Feeds Self With Hands	0.77 (0.53–1.12)	1.48 (0.92–2.36)	1.43 (0.85–2.41)	0.95 (0.59–1.53)
Uses Fork/Spoon With Assistance	1.08 (0.71–1.65)	3.01 (1.81–5.00)*	3.99 (2.18–7.31)*	1.76 (1.05–2.95)*
Uses Fork/Spoon Independently	0.77 (0.43–1.38)	3.51 (2.06–5.98)*	5.26 (2.89–9.56)*	2.83 (1.60–5.01)*

^1^The class II deletion subtype is the reference subtype for the hazard ratio

^2^There was a significant interaction between time and molecular subtype (data not shown), a violation of the Cox proportional hazard assumption

^3^Sex was a significant covariate at *p* < .05 (data not shown) and indicated that at any given time, females were more likely to achieve the skill than males

^4^Recall was a significant covariate at *p* < .05 (data not shown) but the expanded model did not have a significant impact on the hazard ratios (original hazard ratios reported)

^*^Hazard ratio significant at *p* < .05

^†^Data is 100% censored for the reference subtype (class II deletion) or the comparison subtype so a hazard ratio could not be calculated

Likewise, when comparing deletion negative individuals, results from Table 2 suggest that *UBE3A* and ImpD subtypes have a more similar developmental trajectory, developing most skills earlier than class II deletion (significant in 23 and 21 out of 29 testable skills respectively), while the developmental trajectory for UPD was only significantly different from the class II deletion subtype in 14 out of 29 testable skills. Overall, hazard ratios for deletion-negative subtypes were statistically significantly greater than 1 in multiple developmental domains, indicating that individuals without deletion had a higher probability of achieving these skills than individuals with a class II deletion. Although deletion-positive individuals are unlikely to achieve many daily living skills, deletion-negative individuals are likely to eventually develop some of these skills (e.g., washing hands with assistance and using utensils).

As noted in Table 2, there was evidence that the proportional hazards assumption was violated for all six gross motor skills and one fine motor skill (“hold object”). Further analysis indicated that the hazard ratio comparing class II deletion to ImpD was most often driving significant findings. Time-dependent coefficients were examined by analyzing changes in the slope of the Cox regression coefficient over time to determine appropriate time intervals. Findings indicated that the hazard ratios tend to be higher and more often significant compared to the values provided in Table 2, until approximately the age at which the ImpD subtype reached a 50% probability of acquiring the skill. After that age, the hazard rates for ImpD become more similar to those of class II deletion (i.e., hazard ratios approached 1).

Sex was only a statistically significant covariate for toileting skills (*p* < 0.05, model results not shown), such that females had a higher probability of achieving toileting skills compared to males at each age.

Out of 30 skills, recall time was found to be a significant covariate only for “uses toilet without reminder” (*p* < 0.05, model results not shown), suggesting a possible recall bias. Caregivers were more likely to report a younger age of acquisition for this skill when there was a longer time interval between when this skill was acquired and when the caregiver answered this question on the questionnaire. However, the hazard ratios for the expanded model that included recall time (data not shown) were within the confidence intervals of the original model, indicating that the impact of potential recall bias was small and indistinguishable from sampling variability and therefore, the original model results were retained. Sample sizes for censored (i.e., failing to achieve milestone/skill within the observation window) and uncensored (i.e., achieving the milestone/skill within the observation window) cases are provided in Table 3.

**Table 3:** Number of Uncensored and Censored Individuals for Developmental Skills by Molecular Subtype

	SUBTYPES									
	**Class I Deletion**		**Class II Deletion**		***UBE3A* Mutation**		**Imprinting Defect**		**Uniparental Disomy**	
	U	C	U	C	U	C	U	C	U	C
**Gross motor skills**										
Unsupported Sit	65	0	88	1	29	0	20	0	26	0
Commando Crawl	49	19	72	23	19	10	12	8	21	6
Four-Point Crawl	48	21	75	21	27	3	18	3	26	2
Pulls to Stand	57	10	91	5	29	1	20	1	26	2
Walks With Support	63	8	91	7	27	1	22	0	28	0
Walks Independently	45	27	75	25	30	1	21	0	26	3
**Fine motor skills**										
Holds Small Object	59	4	87	2	24	0	17	2	22	0
Reaches for Object	59	0	83	1	26	0	18	0	22	0
Transfers Object Hand to Hand	59	1	80	4	23	0	15	0	15	1
Uses Pincer Grasp	50	20	75	11	24	1	19	1	23	1
**Expressive language skills**										
Gestures/Points for Wants	46	22	65	25	27	1	21	0	26	2
Use of Manual Signs	29	41	40	59	26	1	20	0	25	2
Single Words	19	50	30	67	21	5	16	5	20	9
**Toileting**										
Uses Toilet When Placed	20	28	36	39	13	10	16	1	14	9
Uses Toilet When Reminded	2	46	9	68	10	14	12	5	9	12
Uses Toilet Without Reminder	1	47	5	75	8	18	11	7	9	14
**Dressing**										
Can Remove Some Clothes	41	8	65	13	24	1	19	0	18	1
Can Remove All Clothes	13	40	26	56	17	9	16	3	16	7
Can Put on Some Clothes	6	47	21	65	17	10	16	3	10	11
Can Fully Dress Except Shoes	1	52	4	82	7	20	10	9	1	22
Can Fully Dress Including Shoes	0	53	2	83	7	20	5	14	3	22
**Hygiene**										
Washes Hands With Assistance	9	39	39	41	21	2	16	1	17	5
Washes Hands Independently	3	49	9	74	17	6	13	6	11	13
Bathes With Assistance	3	50	5	81	14	12	8	10	5	19
Bathes Independently	0	53	0	85	3	24	3	16	2	23
Brushes Teeth With Assistance	3	50	10	74	16	11	13	6	14	9
Brushes Teeth Independently	1	51	2	80	8	19	6	13	3	22
**Feeding**										
Feeds Self with Hands	45	1	73	1	24	0	18	0	22	0
Uses Fork/Spoon With Assistance	37	12	53	21	23	0	15	0	20	1
Uses Fork/Spoon Independently	18	32	33	48	24	2	18	0	19	5

Note. *U* Uncensored, *C* Censored

### Age of acquisition for different milestones

The ages (in months) at which there is a 5%, 50%, and 95% probability of acquiring a given skill among participants with each molecular subtype are shown in Table 4 and Figs. 2, 3, 4, 5 and 6. Across all molecular subtypes, fine motor skills such as holding an object were achieved during the first 3.5 years of life while gross motor skills such as walking with support were achieved within the first five years of life. Skills such as pincer grasp showed greater variability in the ages at which they were likely to be achieved even within the same molecular subtype. For example, individuals with UPD and class II deletion were likely to achieve the pincer grasp fine motor skill between ages 10–180 months (a range of 170 months). Daily living skills such as toileting, feeding, and hygiene skills also tended to have substantial variability in the ages at which the skills were likely to be achieved across molecular subtypes.

**Table 4:** Ages in Months and Probability of Achieving Developmental Skills by Molecular Subtype

	Subtypes														
	**Class I Deletion**			**Class II Deletion**			***UBE3A* Mutation**			**Imprinting Defect**			**Uniparental Disomy**		
	**5%**	**50%**	**95%**	**5%**	**50%**	**95%**	**5%**	**50%**	**95%**	**5%**	**50%**	**95%**	**5%**	**50%**	**95%**
**Gross motor skills**															
Unsupported Sit	5	11	24	5	10	24	4	8.5	16	4	8.5	14	5	10	22
Commando Crawl	7	20	ǂ	7	17	ǂ	7	18	ǂ	7	19	ǂ	7	16	ǂ
Four-Point Crawl	10	24	ǂ	10	24	ǂ	9.5	18	ǂ	10	19	ǂ	9.5	18	ǂ
Pulls to Stand	11	24	144	10	22	48	9	16	29	9	18	32	10	21	43
Walks With Support	13	30	61	12	26	54	11	19	35	11	17	30	12	23	42
Walks Independently	21	47	ǂ	20	40	ǂ	17	28	48	15	23	36	17	31	60
**Fine motor skills**															
Holds Small Object	3	9	42	3	9	42	3	8	24	3	9	30	3	9	30
Reaches for Object	3	9	26	3	9	32	2	6	16	2	7	18	3	9	26
Transfers Object Hand to Hand	5	14	48	5	16	84	4	12	36	4	11	30	5	17	ǂ
Uses Pincer Grasp	10	34	ǂ	10	28	180	9	24	108	9	24	96	10	27	180
**Expressive language skills**															
Gestures/Points for Wants	13	46	ǂ	13	39	ǂ	9	24	70	8	22	48	12	30	144
Use of Manual Signs	22	90	ǂ	22	96	ǂ	15	25	60	16	30	90	17	35	120
Single Words	23	†	ǂ	21	†	ǂ	12	34	ǂ	14	42	ǂ	13	41	ǂ
**Toileting**															
Uses Toilet When Placed	36	102	ǂ	30	84	ǂ	30	76	ǂ	29	54	96	30	76	ǂ
Uses Toilet When Reminded	133	†	ǂ	72	†	ǂ	42	113	ǂ	36	60	143	42	120	ǂ
Uses Toilet Without Reminder	192	†	ǂ	96	†	ǂ	52	132	ǂ	42	72	192	48	118	ǂ
**Dressing**															
Can Remove Some Clothes	13	49	276	13	48	276	12	44	126	11	36	108	12	46	144
Can Remove All Clothes	42	†	ǂ	30	156	ǂ	26	78	ǂ	24	59	132	24	66	171
Can Put on Some Clothes	67	†	ǂ	54	168	ǂ	30	84	ǂ	26	66	120	36	96	ǂ
Can Fully Dress Except Shoes	168	†	ǂ	96	†	ǂ	60	132	ǂ	57	96	ǂ	96	†	ǂ
Can Fully Dress Including Shoes	*	†	ǂ	132	†	ǂ	58	†	ǂ	58	†	ǂ	66	†	ǂ
**Hygiene**															
Washes Hands With Assistance	45	†	ǂ	30	95	ǂ	1	54	108	1	45	96	24	60	144
Washes Hands Independently	81	†	ǂ	66	†	ǂ	37	78	ǂ	39	81	ǂ	40	96	ǂ
Bathes With Assistance	84	†	ǂ	76	†	ǂ	36	90	ǂ	46	120	ǂ	57	†	ǂ
Bathes Independently	*	†	ǂ	*	†	ǂ	70	†	ǂ	68	180	ǂ	77	†	ǂ
Brushes Teeth With Assistance	72	†	ǂ	54	†	ǂ	24	72	ǂ	24	78	ǂ	24	78	ǂ
Brushes Teeth Independently	168	†	ǂ	168	†	ǂ	45	168	ǂ	45	168	ǂ	57	†	ǂ
**Feeding**															
Feeds Self With Hands	7	24	84	7	18	60	6	14	48	6	15	48	7	20	72
Uses Fork/Spoon With Assistance	16	54	ǂ	18	60	ǂ	13	30	72	12	29	60	16	47	108
Uses Fork/Spoon Independently	35	142	ǂ	30	120	ǂ	18	48	132	18	43	96	20	54	156

^*^ The highest probability of skill achievement was less than 5%

^†^ The highest probability of skill achievement was less than 50%

ǂ The highest probability of skill achievement was less than 95%

Deletion-positive individuals developed skills at a later age range than the deletion-negative individuals. Results in Table 4 also indicate a wider range of ages for skill acquisition among deletion-positive individuals compared to deletion-negative individuals for most skills with the exception of “Reaches for Object”, “Uses Pincer Grasp”, and “Feeds Self With Hands” where the age range of skill acquisition among those with UPD was comparable to that for those with a deletion.

## Discussion

Using data from a sample of 261 individuals in the AS Natural History study, we examined the attainment of developmental milestones and daily living skills in individuals with AS. Although previous studies have demonstrated that individuals with AS have developmental delays [15, 16, 21, 22], this is the first study to examine the probability and age at which a range of developmental milestones and daily living skills are acquired among individuals with AS due to different molecular etiologies.

We found significant variability in the probability of achieving different skills at various ages across AS subtypes. Of note, some “pre-walking” skills such as commando crawl and four-point crawl were never achieved by some of these individuals despite their ability to achieve higher-level skills such as walking with support. Overall, fine and gross motor skills such as holding and reaching for small objects, sitting, and walking with support had a higher probability of being achieved within a fairly narrow range of ages. On the other hand, many daily living skills such as toileting and feeding, had a lower probability of being acquired and exhibited greater variability in the age at which they were acquired. In addition, independence in performing some daily living skills such as brushing teeth, bathing, and dressing themselves fully were found to be very challenging, with only a small minority of the participants achieving these skills, especially among deletion-positive individuals. Factors such as seizures, access to early intervention services, earlier age of diagnosis, and the availability of new disease-modifying therapies may affect the age at which these milestones are achieved and should therefore be re-examined in future studies.

Additionally, consistent with prior literature, deletion-positive individuals were more delayed (i.e., achieved skills at older ages) and typically had a wider range in age of skill acquisition compared to non-deletion individuals [15, 16, 24, 26]. Deletion-positive individuals also achieved fewer self-care skills such as toileting, using utensils, and washing hands compared to non-deletion individuals. Among deletion-positive individuals, there was no difference in developmental profile of class I and class II deletions for most skills. The developmental profiles of individuals with *UBE3A* pathogenic variants and ImpD were also quite similar for most skills. The developmental profiles of individuals with UPD generally fell in between individuals with class II deletions and *UBE3A* pathogenic variants. These findings are not consistent with prior studies, which have found that individuals with UPD and ImpD have similar developmental profiles and are considered to be least affected developmentally [26]. Given the small sample sizes, the differences between the non-deletion molecular subtypes should be interpreted with caution.

Similarly, it is important to further explore the violation of the proportional hazard model assumption, specifically when comparing the hazard rates of ImpD to class II deletion. This exploration will help determine whether this violation is an artifact of the small sample size or an indication that after a certain age, individuals with ImpD who have not yet attained specific gross and fine motor skills tend to have a developmental profile more similar to deletion-positive individuals for these skills.

Sex differences were only evident in toileting skills, with females demonstrating a higher probability of achieving these skills compared to males. These results are consistent with findings in the neurotypical population where girls have been noted to have better bladder control attributed to their shorter urethra and heightened bodily awareness compared to boys [29]. It is also possible that parents may be attempting to initiate toilet training earlier for girls than for boys [30].

The failure to achieve daily living skills creates significant functional impairment and limits the ability of individuals to function independently, consistent with needing lifelong support and assistance [31–33]. These functional impairments may be sources of stress for caregivers and affect family quality of life [34]. It is therefore critical that ongoing interventions target a variety of daily living skills. Daily living skills should be broken down into smaller, more achievable tasks that can then be taught using behavioral techniques such as shaping, chaining, and discrete trial instruction [35, 36]. For example, in terms of toileting, the results of the current study suggest that individuals with AS are more likely to use the toilet when placed there rather than doing so independently. As such, working on regular or scheduled toilet visits with positive reinforcement may be appropriate strategies when toilet training individuals with AS [37, 38].

There are important clinical implications arising from our study. Our study provides clinicians and families with a useful tool to help provide more accurate developmental prognosis for children with AS, to monitor the developmental progress of these children, and for setting appropriate therapeutic and educational goals. If a child fails to make adequate developmental progress with intensive therapies, ruling out other medical concerns (e.g., seizures, co-existing genetic disorders) is critical. In addition, given the level of delay in acquisition of skills, individuals with AS should receive intensive physical, occupational, and speech-language therapy beginning early in life and continuing through adulthood. Teaching of functional daily living skills should be an important component of Individualized Education Plans for children with AS.

Although this study has many strengths, including a relatively large sample size for a rare disorder, we acknowledge some notable limitations. The use of caregiver report to gather information on the acquisition of developmental data may not be reliable. Caregivers are more likely to accurately recall some milestones compared to others because they are more meaningful to them (e.g., walking independently versus reaching) [39, 40]. In addition, when caregivers were asked to recall when a specific skill was achieved with assistance, the definition of ‘with assistance’ was not provided and could have been interpreted differently by parents. Although we assessed the data collected for recall bias based on age of recall and found limited evidence, prior studies have found parental report to be less accurate than assessments administered and scored by clinicians [41]. The presence of seizures could influence the age of acquisition of developmental milestones. However, due to the limited sample size of individuals without seizures in each genotype, and the unknown seizure status of several participants, we were unable to assess the potential effect of seizures on the development of various skills. Additional limitations are the relatively small sample sizes for some of the molecular subtypes used to calculate hazard rates and ratios, and the failure of all participants to achieve some of these skills during the data collection timeframe for many skills (i.e., highly censored data). Although continued longitudinal tracking of individuals with AS may decrease the proportion of censored data, if some skills are only achieved by a very small proportion of the AS population, censored data are unavoidable.

There was some evidence of violation of the proportional hazard assumption for the gross and fine motor skills. However, this mainly affected the point estimates of the hazard ratios for the ImpD and class II deletion comparisons. Notably, the pattern of results for these skills was consistent with the pattern observed for the other skills that did not violate the proportional hazard assumption, namely, that the hazard ratios comparing ImpD to class II deletion were typically greater than 1, indicating individuals with ImpD tended to acquire skills at a faster rate compared to those with class II deletion. In addition, our study focused primarily on the ages at which developmental milestones and adaptive skills were acquired. We did not gather additional information on how individuals with AS were using these specific skills in their daily life, whether the ability to use these skills was influenced by sensory or behavior challenges or lack of motivation, or whether there was any loss of skills over time. Moreover, the expressive language domain focused only on the age of acquisition of gestures, manual signs, and verbal speech; it did not include information on the use of augmentative and alternative communication systems. Future studies with a larger sample should not only assess whether an individual has acquired a skill but also whether they are able to effectively use a skill in their daily life, and whether regression is seen in any of the skills (especially as individuals age). Predictors of variability in developmental skills within molecular subtypes such as age of diagnosis, age of access to services, and presence of seizures should also be examined to better inform clinical care.

## Conclusions

Using data from the AS Natural History study, we analyzed the probability and age at which various developmental milestones and daily living skills in individuals with AS across molecular subtypes were acquired. Results demonstrate significant developmental delays and functional challenges in AS and suggest the need for intensive interventions beginning early in life.

### Supplementary Information

Supplementary Material 1.

## Data Availability

The datasets generated and/or analyzed during the current study are available in the Linking Angelman and Dup15q Data for Expanded Research (LADDER) database https://laddertotreatment.org/

## Abbreviations

AS: Angelman syndrome
UPD: Uniparental disomy
ImpD: Imprinting defect
CPH: Cox proportional hazard
