# Supplementary material for: Developmental milestones and daily living skills in individuals with Angelman syndrome
Source: J Neurodev Disord. 2024 Jun 15;16:32. doi: 10.1186/s11689-024-09548-7 (PMC11179294; doi:10.1186/s11689-024-09548-7)
Supplement: Supplementary file 1 — Supplementary Material 1. [file 11689_2024_9548_MOESM1_ESM.docx]

**Supplementary Materials**

**Developmental Milestones**

**Skills analyzed in the current study*

**Gross Motor Development:**  No Change Since Last Visit

1. Rolls front to back  Yes at age _____ months  No  Unknown
2. Rolls back to front  Yes at age _____ months  No  Unknown
3. Sits unsupported*  Yes at age _____ months  No  Unknown
4. Commando Crawl*  Yes at age _____ months  No  Unknown
5. Four-Point Crawl*  Yes at age _____ months  No  Unknown
6. Pulls self to stand*  Yes at age _____ months  No  Unknown
7. Walks with support*  Yes at age _____ months  No  Unknown
8. Walks independently*  Yes at age _____ months  No  Unknown
9. Navigate stairs up  Yes at age _____ months  No  Unknown
10. Navigate stairs down stairs  Yes at age _____ months  No  Unknown
11. Pedals tricycle  Yes at age _____ months  No  Unknown

**Fine Motor Development:**  No Change Since Last Visit

1. Bring hands together  Yes at age _____ months  No  Unknown
2. Holds small object*  Yes at age _____ months  No  Unknown
3. Reaches for object*  Yes at age _____ months  No  Unknown
4. Transfers object one hand to other*  Yes at age _____ months  No  Unknown
5. Uses pincer grasp*  Yes at age _____ months  No  Unknown
6. Finger feeds  Yes at age _____ months  No  Unknown

**Expressive Language Development:**  No Change Since Last Visit

1. Smiles responsively  Yes at age _____ months  No  Unknown
2. Makes sounds of pleasure  Yes at age _____ months  No  Unknown
3. E.g. coos (ooh/aah)
4. Babbles(single sounds  Yes at age _____ months  No  Unknown e.g.. m, b)
5. Gestures/points for wants*  Yes at age _____ months  No  Unknown
6. Use of Manual Signs*  Yes at age _____ months  No  Unknown

If yes, number of signs used:  0-5  6-10  11-30  >30

1. Single words*  Yes at age _____ months  No  Unknown

If yes, number of single words used:  0-5  6-10  11-30  >30

1. Phrase Speech (2-3 word phrases)  Yes at age _____ months  No  Unknown

If yes, examples of phrases _____________________________________________

**Receptive Language Development:**  No Change Since Last Visit

1. Fix/Follow (past midline)  Yes at age _____ months  No  Unknown
2. Follows one step commands when accompanied with gesture  Yes at age _____ months  No  Unknown
3. Follows one step commands without gesture Yes at age _____ months  No  Unknown

**Life Skills**

**Toileting: ** No change since last visit

1. Uses toilet when placed there; stays dry & unsoiled as long as taken to toilet at regular intervals (time training)*  Yes at age _____ months  No  Unknown

1. Uses toilet when reminded to do so; stays dry & unsoiled as long as reminded at regular intervals, does not need assistance with mechanics of toileting*  Yes at age _____ months  No  Unknown
2. Uses toilet at regular intervals without being reminded*  Yes at age _____ months  No  Unknown

**Dressing:**  No change since last visit

1. Can remove some, but not all, clothes*  Yes at age _____ months  No  Unknown
2. Can remove all clothes*  Yes at age _____ months  No  Unknown
3. Can put on some, but not all clothes*  Yes at age _____ months  No  Unknown
4. Can fully dress self, but not able to put on shoes*  Yes at age _____ months  No  Unknown
5. Can full dress self, including shoes (okay if slip-ons, Velcro)*  Yes at age _____ months  No  Unknown

**Hygiene:**  No change since last visit

1. Washes hands with assistance (requires help in turning on/off water or soaping hands)*  Yes at age _____ months  No  Unknown
2. Washes hands independently (purposeful hand-washing, not simply playing with water) without assistance other than reminder*  Yes at age _____ months  No  Unknown
3. Bathes/showers self with assistance such as regulating water temperature*  Yes at age _____ months  No  Unknown
4. Bathes/showers self independently (no assistance other than reminder to do so)*  Yes at age _____ months  No  Unknown
5. Brushes teeth with assistance (caretaker readies toothbrush and monitors at end)*  Yes at age _____ months  No  Unknown
6. Brushes teeth independently*  Yes at age _____ months  No  Unknown

**Feeding:**  No change since last visit

1. Feeds self with hands*  Yes at age _____ months  No  Unknown
2. Feeds self with fork/spoon with assistance if parent uses hand-over-hand technique*  Yes at age _____ months  No  Unknown
3. Feeds self with fork/spoon independently without assistance other than caretaker cutting food into pieces*  Yes at age _____ months  No  Unknown
